# Increased cancer risks for relatives of very early-onset breast cancer cases with and without BRCA1 and BRCA2 mutations

**DOI:** 10.1038/sj.bjc.6605876

**Published:** 2010-09-07

**Authors:** G S Dite, A S Whittemore, J A Knight, E M John, R L Milne, I L Andrulis, M C Southey, M R E McCredie, G G Giles, A Miron, A I Phipps, D W West, J L Hopper

**Affiliations:** 1 Centre for Molecular, Environmental, Genetic and Analytic Epidemiology, The University of Melbourne, Level 1, 723 Swanston Street, Melbourne, Carlton VIC 3053, Australia; 2 Department of Health Research and Policy, Stanford University School of Medicine, Stanford, CA, USA; 3 Samuel Lunenfeld Research Institute, Mount Sinai Hospital, Toronto, Ontario, Canada; 4 Dalla Lana School of Public Health, University of Toronto, Toronto, Ontario, Canada; 5 Northern California Cancer Center, Fremont, CA, USA; 6 Departments of Molecular Genetics and Laboratory Medicine and Pathobiology, University of Toronto, Ontario, Canada; 7 Department of Pathology, The University of Melbourne, Melbourne, Australia; 8 Department of Preventive and Social Medicine, University of Otago, Otago, New Zealand; 9 Cancer Epidemiology Centre, Cancer Council Victoria, Melbourne, Australia; 10 Dana-Faber Cancer Institute, Boston, MA, USA

**Keywords:** brain cancer, breast cancer, lung cancer, ovarian cancer, prostate cancer, urinary cancers

## Abstract

**Background:**

Little is known regarding cancer risks for relatives of women with very early-onset breast cancer.

**Methods:**

We studied 2208 parents and siblings of 504 unselected population-based Caucasian women with breast cancer diagnosed before age 35 years (103 from USA, 124 from Canada and 277 from Australia), 41 known to carry a mutation (24 in BRCA1, 16 in BRCA2 and one in both genes). Cancer-specific standardised incidence ratios (SIRs) were estimated by comparing the number of affected relatives (50% verified overall) with that expected based on incidences specific for country, sex, age and year of birth.

**Results:**

For relatives of carriers, the female breast cancer SIRs were 13.13 (95% CI 6.57–26.26) and 12.52 (5.21–30.07) for BRCA1 and BRCA2, respectively. The ovarian cancer SIR was 12.38 (3.1–49.51) for BRCA1 and the prostate cancer SIR was 18.55 (4.64–74.17) for BRCA2. For relatives of non-carriers, the SIRs for female breast, prostate, lung, brain and urinary cancers were 4.03 (2.91–5.93), 5.25 (2.50–11.01), 7.73 (4.74–12.62), 5.19 (2.33–11.54) and 4.35 (1.81–10.46), respectively. For non-carriers, the SIRs remained elevated and were statistically significant for breast and prostate cancer when based on verified cancers.

**Conclusion:**

First-degree relatives of women with very early-onset breast cancer are at increased risk of cancers not explained by BRCA1 and BRCA2 mutations.

Familial aggregation of disease on a population basis is measured by the increase in risk for closely related relatives of an affected person. Having one or more close relatives with breast cancer is an important and well-established risk factor for that disease, and the magnitude of risk varies depending on the number of, and relationship to, affected relatives and the ages at which the relatives were diagnosed. A meta-analysis (Pharoah *et al*, 1997) and a large pooled analysis (Collaborative Group on Hormonal Factors in Breast Cancer, 2001) found that, on average, women have a two-fold increased risk if they have a first-degree relative with breast cancer, a three-fold increased risk if they have two affected first-degree relatives and a four-fold increased risk if they have three affected first-degree relatives. These increased risks are most pronounced for younger women and women with at least one affected relative diagnosed at an early age.

As these studies have generally studied breast cancer across all ages, little is known regarding the breast cancer risks for relatives of women with *very* early-onset breast cancer, such as those diagnosed before the age of 35 years, let alone their risks of other cancers except ovarian cancer (Anderson *et al*, 2000; Dong and Hemminki, 2001; Hemminki and Granstrom, 2004). There is no consistent evidence for an increased risk of cancer at other specific sites, although some studies found a risk of cancer other than breast cancer (Goldgar *et al*, 1994; Anderson *et al*, 2000).

We have expanded previous work from an Australian study (Dite *et al*, 2003) by incorporating data from two further studies carried out in North America. We used data from the Breast Cancer Family Registry (Breast CFR), a large international consortium that has enrolled more than 13 000 breast cancer families (John *et al*, 2004), to study cancer risks for close relatives of women (index cases) who had very early-onset breast cancer and had been identified through population-based cancer registries, a large proportion of whom had been screened for mutations in BRCA1 and BRCA2. We estimated risks of breast and other cancers for groups of relatives defined by the BRCA1 and/or BRCA2 mutation status of their index case.

## Materials and methods

### Subjects

Index cases were women with breast cancer ascertained through population-based cancer registries and recruited to the Breast CFR in San Francisco (USA), Ontario (Canada) and Melbourne and Sydney (Australia) (McCredie *et al*, 1998; John *et al*, 2004); see also the Supplementary Material. Index cases were restricted to those diagnosed before the age of 35 years with an incident, first primary breast cancer who self-reported being Caucasian and for whom information on their biological parents and full siblings was available.

In San Francisco, 185 (61%) of 301 index cases participated (24 were deceased, 6 had physician-reported contraindications for contact, 1 was too ill, 20 could not be located, 53 declined to participate and 8 did not respond). In Ontario, 226 (62%) of 362 index cases participated (for 19 the treating physician refused permission to contact, for 7 the treating physician could not be located, 4 were deceased, 11 could not be contacted, 30 refused and 65 did not respond). In Melbourne and Sydney, a breakdown of participation for cases diagnosed before age 35 years was not available, but for the 1208 identified index cases under the age of 40 years at diagnosis, 856 (71%) participated (for 102 the treating physician refused permission to contact, for 10 the treating physician did not respond, 19 were deceased, 35 had moved, 168 declined to participate and 18 did not respond).

After restricting index cases to those who self-identified as Caucasian and for whom there was cancer history information on their biological parents and siblings, a total of 504 index cases (103 from San Francisco, 124 from Ontario, and 277 from Melbourne and Sydney) were included in this study.

### Family cancer histories

All index cases completed the same Breast CFR family history questionnaire (John *et al*, 2004) that included an enumeration of at least all first-degree relatives and information on known cancer histories. Recruitment of living parents and adult siblings of the index cases was sought wherever possible. Participating relatives then provided cancer history information regarding themselves and their relatives. A substantial proportion of the information collected for first-degree relatives was provided independently by the relatives themselves. Documented verification of reported cancers (through pathology reports, cancer registries and medical records) was sought wherever possible. Overall, 50% of reported cancers were verified, 63% of breast cancers and 43% of non-breast cancers. Non-melanoma skin cancers were excluded from analyses. All participants provided written informed consent before participation and all studies were approved by the relevant local ethics committees.

### Imputation of missing family data

For each index case, the data required on each of their parents and siblings for these analyses were: relationship to the index case, date of birth, vital status, age at interview or death, and for those who had had cancer, the site and age at diagnosis. For some relatives, one or more of the above data items were missing and could not be calculated directly from known data. A total of 48 relatives had missing date of birth, 3 deceased relatives had missing age at death and 13 with cancer had missing age at diagnosis. In these instances, data were imputed iteratively using a variation of a previously developed protocol (Dite *et al*, 2003); see Supplementary Material for more details. A further 10 had missing vital status and were censored at age 20.

### BRCA1 and BRCA2 mutation testing

For the purpose of this study, mutations were protein-truncating or missense mutations classified deleterious as by the Breast Cancer Information Core (National Human Genome Research Institute, 2002). Details of testing are given in the Supplementary Material. BRCA1 and BRCA2 mutation testing was conducted for 84% and 82%, respectively, of all index cases, and of all index cases with a first-degree family history of breast cancer.

To estimate the number of index case mutation carriers not identified by testing, we multiplied the number tested by each method by 1 – proportion of the coding region of the gene covered (0.9 for sequencing, two-dimensional gel electrophoresis and heteroduplex; 0.8 for whole gene PTT; 0.6 for limited gene PTT; and 0.1 for founder mutation screening) and multiplied by the mutation frequency (0.15 and 0.05 for those with and without an affected first-degree relative, respectively). For index cases for whom no testing was conducted, the number of missed mutations was estimated by multiplying the number not tested by the mutation frequency above.

Of the 374 tested index cases for whom no mutation was found, we estimated that 4.7 BRCA1 and 7.3 BRCA2 carriers were not detected. Of the 89 index cases not tested, we estimated there were 5.3 BRCA1 and 5.6 BRCA2 carriers not detected. Therefore, we estimated that 5% of the index cases not tested, or found not to carry a mutation, were carriers.

### Cancer incidence

For each cancer site, population-based cancer incidence rates specific for age (in 5-year groupings), year of birth (in 5-year groupings), gender and country were obtained for: 1925–1985 in Connecticut, USA (http://seer.cancer.gov); for 1965–2001 in Ontario, Canada (Ontario Cancer Registry, 2004); and for 1983–2001 in Australia (http://www.aihw.gov.au). For Ontario and Australia, cancer incidences were not available before 1965 and 1983, respectively. These incidences were extrapolated from the available data by calculating the known Ontario and Australian incidences as a proportion of the Connecticut rates and applying this proportion to the Connecticut data for the missing years. Specific cancer sites chosen for inclusion in this study were those which are known to be common (colorectal, brain, cervix, lung, stomach, urinary) and/or of particular interest in relation to breast cancer (ovary, prostate). Urinary cancers were defined as cancers of the kidney, bladder, ureter and renal pelvis.

### Statistical methods

We estimated the standardised incidence ratio (SIR) by comparing the observed number of affected in a group of relatives to those expected in the general population. The age-specific cumulative cancer incidence was estimated by Kaplan–Meier product-limit survival functions in which failure time was age at cancer diagnosis. Unaffected relatives were censored at age at completion of family history questionnaire if alive, age at death if deceased or age 20 years if vital status was unknown. Cumulative incidence was defined as the complement of the Kaplan–Meier survival function. All statistical analyses were performed with STATA version 9, (2005) (Stata Corporation, College Station, TX, USA). All statistical tests were two-sided and, following convention, *P*-values <0.05 were considered nominally statistically significant.

## Results

Table 1 shows the centre-specific distribution of the index cases by age at diagnosis, family history of breast cancer and BRCA1 and BRCA2 mutation status. The mean age at diagnosis of the index cases was 31.3 years (s.d. 2.8 years). Of these, 41 carried a mutation (24 in BRCA1, 16 in BRCA2 and 1 in both genes).

The 504 index cases reported having 504 fathers, 504 mothers, 602 full brothers and 598 full sisters with a combined total of 99 648 person-years of observation. The mean (s.d.) person-years was 59.8 (9.4) for fathers, 58.1 (8.5) for mothers, 33.5 (8.1) for brothers and 33.6 (7.4) for sisters.

Table 2 shows that the SIRs for breast cancer were elevated overall and in each of the subgroups defined by relationship to the index case, centre or testing status of the index case. The SIR for relatives of index cases with mutations in BRCA1 were 3.3 times that for relatives of index cases who tested negative (*P*=0.003) and 2.5 times that for relatives of index cases who were not tested (*P*=0.05). For relatives of index cases with mutations in BRCA2, the SIR was 3.1 times that for relatives who tested negative (*P*=0.02) and 2.4 times that for relatives who were not tested (*P*=0.1).

**Figure 1:**
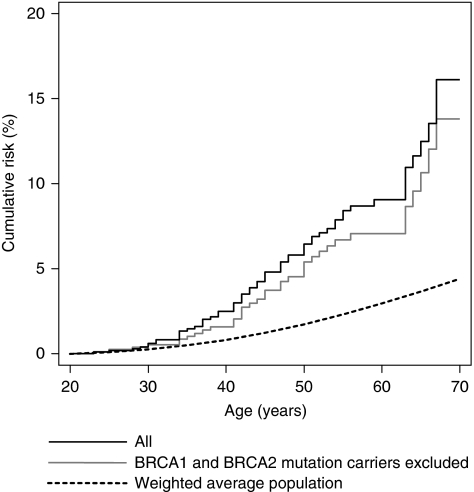
Cumulative risk of breast cancer for mothers and sisters combined.

Overall, the breast cancer SIR was 1.7 times greater for sisters than mothers (*P*=0.1). For relatives of the index cases not found to carry mutations in BRCA1 and BRCA2, the SIR was 1.3 times greater for sisters than mothers (*P*=0.5). Although the SIRs for sisters and mothers were 30 and 15% lower, respectively, for the relatives of the index cases who were not found to carry a mutation compared with all index cases, they were both elevated compared with the general population; see Figure 1.

When analyses were restricted to the 36 verified breast cancers, the SIR for mothers and sisters was 3.04 (95% CI 2.20–4.20) overall. For relatives of the index cases found to carry mutations in BRCA1 and BRCA2, the SIRs were 8.21 (95% CI 3.42–19.72) and 7.51 (95% CI 2.42–23.29), respectively, whereas for relatives of index cases who were not found to be a carrier or had not been tested, the SIRs were 2.58 (95% CI 1.71–3.88) and 2.62 (95% CI 1.09–6.28), respectively.

Table 3 shows that the SIRs for all non-breast cancers (excluding non-melanoma skin cancer) were elevated both overall and for each of the subgroups, for brothers, the subgroup with the least observed cancers, the SIR was only marginally elevated. The SIR for cancers other than breast cancer for fathers and brothers combined was not different to that for mothers and sisters combined (*P*=0.5). The SIR for brothers and sisters combined was 1.6 times that for fathers and mothers combined (*P*=0.03). For the male relatives, the SIR for fathers was not different to that for brothers (*P*=0.9), whereas for female relatives the SIR for sisters was 2.5 times that for mothers (*P*<0.01).

For subgroups defined by mutation status of the index case, the non-breast cancers SIR for relatives of the index cases who carried BRCA1 mutations was marginally elevated while the SIR for relatives of the index cases who carried BRCA2 mutations was not elevated. Elevated SIRs were seen for relatives of index cases who had been tested and found not to be a carrier or were untested. After limiting analyses to relatives of the index cases not found to carry mutations, the SIRs remained elevated for all subgroups except for brothers and marginally so for mothers. The SIR for brothers and sisters combined was higher than for fathers and mothers combined (*P*=0.03) and the SIR for sisters remained higher than that for mothers (*P*<0.01).

**Figure 2:**
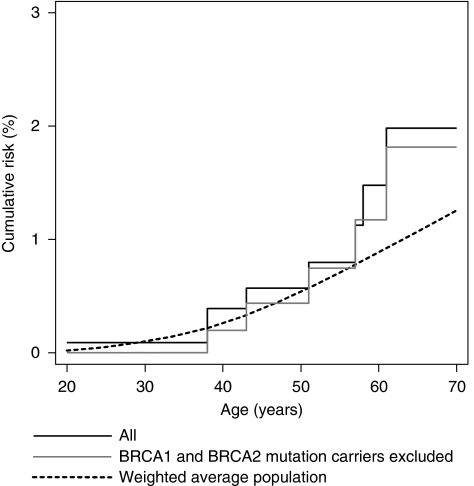
Cumulative risk of ovarian cancer for mothers and sisters combined.

Table 4 shows cancer site-specific SIRs. The ovarian cancer SIR was elevated overall (2.46; 95% CI 1.23–4.92; *P*=0.01) and for mothers and sisters of BRCA1 mutation carriers (*P*<0.001), but not for mothers and sisters of index cases who tested negative or were not tested; see also Figure 2. No ovarian cancers were observed in relatives of BRCA2 mutation carriers.

For prostate cancer, the SIR was substantially elevated overall (6.54; 95% CI 3.71–11.51; *P*<0.001) and within subgroups defined by BRCA2 mutation carrier and testing status (all *P*<0.001). No prostate cancers were observed in relatives of BRCA1 mutation carriers. Using the eight verified prostate cancers only, the SIRs for fathers and brothers remained elevated and were 4.36 (95% CI 2.18–8.72) overall and 3.75 (95% CI 1.56–9.01) for relatives of known non-carriers (both *P*<0.001).

For brain cancer, the SIR was elevated overall (2.79; 85% CI 1.26–6.22; *P*=0.01) and for the subset of relatives of known non-carriers (both *P*=0.01). No relatives of known BRCA1 or BRCA2 carriers were diagnosed with brain cancer. When analyses were restricted to using the four verified brain cancers, the SIRs for relatives of known non-carriers of 1.86 (95% CI 0.70–4.96) overall and 2.34 (95% CI 0.88–6.24) were greater than unity but not significant.

For lung cancer, the SIR was elevated overall (7.04; 95% CI 4.54–10.91; *P*<0.001) and for relatives of index cases who were not tested or known not to be carriers (*P*=0.05 and *P*<0.001, respectively), and for the latter group the SIRs of 5.22 (95% CI 2.72–10.03) for males and 20.34 (95% CI 9.70–42.66) for females were different (*P*=0.007). The SIRs for relatives of BRCA1 and BRCA2 carriers were not elevated. When restricted to the seven verified lung cancers, the SIR was elevated overall (SIR=2.46; 95% CI 1.17–5.17; *P*=0.02), and for males (SIR=2.52; 95% CI 1.13–5.62); *P*=0.02).

For urinary cancers, the SIR was elevated overall (4.45; 95% CI 2.12–9.34; *P*<0.001) and for relatives of known non-carriers (*P*=0.001). No urinary cancers were observed in the relatives of BRCA1 or BRCA2 mutation carriers. When analyses were restricted to using the five verified cancers, the SIR was 3.18 (95% CI 1.32–7.46) overall.

For colorectal cancer, the overall SIR was 0.85 (95% CI 0.48–1.50) while for stomach cancer, the overall SIR was 1.10 (95% CI 0.57–2.11)

## Discussion

Mothers and sisters of women with breast cancer diagnosed before the age of 35 years were at a substantially increased risk of breast cancer, by a factor that remained high even for those whose index cases had been tested and found not to carry mutations in BRCA1 and BRCA2. The risk for sisters was greater than for mothers, consistent with previous studies (Pharoah *et al*, 1997; Collaborative Group on Hormonal Factors in Breast Cancer, 2001; Dite *et al*, 2003). Also consistent with studies of relatives of women with breast cancer at older ages (Goldgar *et al*, 1994; Anderson *et al*, 2000), we found that the parents and siblings were at a 1.5- to 2-fold increased risk of cancers other than breast cancer. The magnitude of this increased risk was little changed when restricted to relatives of the index cases who were tested and found not to carry mutations in BRCA1 and BRCA2.

Overall, first-degree relatives were at increased risks for cancer of the ovary, prostate, lung, brain and urinary tract. The increased risk of ovarian cancer was evident only for the relatives of known BRCA1 mutation carriers, and the increased risk of prostate cancer was evident only for the relatives of known BRCA2 mutation carriers. Although based on small numbers, both these observations are consistent with the literature (Antoniou *et al*, 2003; Willems *et al*, 2008). The increased risks of prostate, brain and urinary cancers for the relatives of known non-carriers could therefore represent real and novel associations.

A potential limitation of all studies of familial risk is the accuracy of data on outcomes for relatives. When comparing with population data, under-reporting of cancers in relatives (false negatives) reduces risk estimates, whereas over-reporting of cancers in relatives (false positives) inflates risk estimates.

A particular strength of the Breast CFR data was the attempt to recruit family members of index cases, with the consequence that the cancer histories of relatives were frequently self-reported or reported by one or more close relatives. By limiting our analyses to first-degree relatives of the index case, any cancer histories that were not self-reported must have been provided by a first-degree relative. Validation studies have shown that, for self-reports, 91% of breast cancers and 79% of cancers at other sites are true positives (Bergman *et al*, 1998) and that for reports by first-degree relatives, 88% of breast cancers and at least 70% of cancers at other sites are true positives (Ziogas and Anton-Culver, 2003) and for all cancers 99% of negative reports were true negatives (Ziogas and Anton-Culver, 2003).

In this study, considerable effort was also made to check the accuracy of the cancer history data and therefore maximise the validity of the results. Wherever possible, reported family cancers were validated using cancer registry data, medical reports and death certificates (John *et al*, 2004).

For the observed cancers in first-degree relatives (see Table 4), 4 of the 8 ovarian cancers, 8 of the 12 prostate cancers, 4 of the 6 brain cancers, 4 of the 6 urinary cancers and 8 of the 20 lung cancers had been confirmed by pathology reports, medical records or death certificates. When analyses were restricted to only using these verified cancers, the SIRs for relatives of known non-carriers remained significantly elevated for prostate cancer, and for lung cancer for subgroups including males (for which the possibility that the lung cancers could be metastasised breast cancers can be ruled out), and were numerically greater than unity for the other cancer groups.

Despite these efforts to collect high-quality data, a potential weakness of our study remains in that there could be over-reporting of cancers in case families. In our overall analyses, some of the non-verified cancers may have been incorrectly reported while in our analyses of verified cancers only, some of the correctly reported but not verified cancers have been excluded. The estimates of SIR from the overall and verified analyses therefore provide upper and lower limits between which the best estimate of the true SIR lies.

We limited index cases to Caucasian women to provide a racially homogeneous (and therefore less genetically heterogeneous) group of families, and obtained relevant population cancer incidence data by using historical cancer registry information so that we could take into account changing cancer-specific incidences over time. In this regard the dotted lines in Figures 1 and 2 are only an indication of the risk for the comparison group. The SIR analyses presented in Tables 2, 3 and 4 represent the ratio of risks adjusted analytically for the fact that the cancer risks for relatives depend on when and where they were living.

A potential weakness of the study is that, although the detection of mutations in BRCA1 and BRCA2 was comprehensive, it was not exhaustive and there is a possibility that some mutations in index cases were missed. There was no systematic difference between those who were tested and were not tested based on family history of breast cancer. We estimated that, at most, we may have tested and misclassified as non-carriers 12 actual mutation carriers (4.7 in BRCA1 and 7.3 in BRCA2), about 3%. If this was the case then the increased breast cancer risk seen in the restricted analysis would predict that we have slightly over-estimated the true breast cancer risk for relatives of non-carriers. This is unlikely to explain the increased cancer risks seen in the restricted analyses.

Our results provide evidence for the existence of familial factors, such as variants in genes other than BRCA1 and BRCA2, which contribute to the increased risks for breast, prostate, lung and/or brain cancers in relatives of women with very early-onset breast cancer. This has implications for gene discovery by focussing attention on families known not to be segregating BRCA1 or BRCA2 mutations with one or more breast cancers diagnosed at a young age as well as one or more of the above cancers. This approach would be similar to those that had pivotal roles in localising BRCA1 through using breast–ovary cancer families (Easton *et al*, 1993), and cloning BRCA2 through studying breast cancer families with male breast cancer (Wooster *et al*, 1995).

## Figures and Tables

**Table 1:** Characteristics of index cases

	**San Francisco**	**Ontario**	**Melbourne and Sydney**	**Total**
	***N* (%)**	***N* (%)**	***N* (%)**	***N* (%)**
*Age at diagnosis*				
20–24 years	5 (5%)	1 (1%)	10 (4%)	16 (3%)
25–29 years	13 (13%)	23 (19%)	57 (21%)	93 (18%)
30–34 years	85 (83%)	100 (81%)	210 (76%)	395 (78%)
				
*Breast cancer family history* a				
None	88 (85%)	107 (86%)	252 (91%)	447 (89%)
One	15 (15%)	15 (12%)	21 (8%)	51 (10%)
Two or more	0 (0%)	2 (2%)	4 (1%)	6 (1%)
				
*BRCA1 mutation testing*				
Not tested	21 (20%)	37 (30%)	27 (11%)	85 (17%)
Negative	74 (72%)	81 (65%)	239 (85%)	394 (78%)
Positive	8 (8%)	6 (5%)	11 (4%)^b^	25 (5%)^b^
				
*BRCA2 mutation testing*				
Not tested	24 (23%)	37 (30%)	29 (10%)	90 (18%)
Negative	77 (75%)	80 (65%)	240 (87%)	397 (79%)
Positive	2 (2%)	7 (6%)	8 (3%)^b^	17 (3%)^b^

a In mothers or sisters of the index case.

b One index case had mutations in both BRCA1 and BRCA2.

**Table 2:** Risk of breast cancer for mothers and sisters of index cases

	**Observed**	**Expected**	**SIR (95% CI)**
Mothers and sisters combined	59	11.83	4.99 (3.86–6.44)
Mothers	48	10.41	4.61 (3.48–6.12)
Sisters	11	1.42	7.73 (4.28–14.00)
San Francisco	15	3.15	4.77 (2.88–7.91)
Ontario	17	2.86	5.95 (3.70–9.58)
Melbourne and Sydney	27	5.83	4.63 (3.18–6.75)
Mothers and sisters of BRCA1-positive index cases	8	0.61	13.13 (6.57–26.26)
Mothers and sisters of BRCA2-positive index cases	5	0.40	12.52 (5.21–30.07)
Mothers and sisters of BRCA1- and BRCA2-negative index cases	36	8.92	4.03 (2.91–5.93)
Mothers and sisters of BRCA1 and BRCA2 untested index cases	10	1.91	5.23 (2.81–9.72)
Mothers of BRCA1- and BRCA2-negative index cases	30	7.77	3.86 (2.70–5.23)
Sisters of BRCA1- and BRCA2-negative index cases	6	1.16	5.19 (2.33–11.54)

Abbreviations: CI=confidence interval; SIR=standardised incidence ratio.

**Table 3:** Risk of non-breast cancer for parents and siblings of index cases^a^

	**Observed**	**Expected**	**SIR (95% CI)**
Parents and siblings	143	72.44	1.97 (1.68–2.33)
Fathers and brothers	79	38.15	2.07 (1.66–2.58)
Mothers and sisters	64	34.29	1.87 (1.46–2.38)
Parents	114	62.34	1.83 (1.52–2.20)
Siblings	29	10.10	2.87 (2.00–4.13)
Fathers	69	33.05	2.09 (1.65–2.64)
Mothers	45	29.30	1.54 (1.15–2.06)
Brothers	10	5.11	1.96 (1.05–3.64)
Sisters	19	4.99	3.80 (2.43–5.96)
San Francisco	41	16.49	2.49 (1.83–3.38)
Ontario	33	17.10	1.93 (1.37–2.72)
Melbourne and Sydney	69	38.86	1.78 (1.40–2.25)
Index case: BRCA1 positive	8	3.94	2.03 (1.02–4.06)
Index case: BRCA2 positive	5	2.75	1.82 (0.76–4.36)
Index case: negative	107	54.46	1.97 (1.63–2.38)
Index case: untested	23	11.40	2.02 (1.34–3.04)
Index case: negative – fathers and brothers	58	28.40	2.04 (1.58–2.64)
Index case: negative – mothers and sisters	49	22.06	1.88 (1.42–2.49)
Index case: negative – parents	83	46.33	1.79 (1.45–2.22)
Index case: negative – siblings	24	8.13	2.95 (1.98–4.41)
Index case: negative – fathers	50	24.34	2.05 (1.56–2.71)
Index case: negative – mothers	33	21.99	1.50 (1.07–2.11)
Index case: negative – brothers	8	4.06	1.97 (0.98–3.94)
Index case: negative – sisters	16	4.07	3.93 (2.41–6.42)

Abbreviations: CI=confidence interval; SIR=standardised incidence ratio.

a Excludes non-melanoma skin cancer.

**Table 4:** Risk of specific cancers for parents and siblings of index cases

	**Observed**	**Expected**	**SIR (95% CI)**
*Ovarian cancer (mothers and sisters)*			
Index case: BRCA1 positive	2	0.16	12.38 (3.10–49.51)
Index case: BRCA2 positive	0	0.11	—
Index case: negative	5	2.45	2.04 (0.85–4.91)
Index case: unknown	1	0.54	1.87 (0.26–13.26)
			
*Prostate cancer (fathers and brothers)*			
Index case: BRCA1 positive	0	0.13	—
Index case: BRCA2 positive	2	0.11	18.55 (4.64–74.17)
Index case: negative	7	1.33	5.25 (2.50–11.01)
Index case: unknown	3	0.26	11.51 (3.71–35.69)
			
*Colorectal cancer (parents and siblings)*			
Index case: BRCA1 positive	1	0.74	1.35 (0.19–9.57)
Index case: BRCA2 positive	1	0.49	2.03 (0.29–14.41)
Index case: negative	8	10.77	0.74 (0.37–1.49)
Index case: unknown	2	2.09	0.96 (0.24–3.83)
			
*Brain cancer (parents and siblings)*			
Index case: BRCA1 positive	0	0.11	—
Index case: BRCA2 positive	0	0.07	—
Index case: negative	5	1.61	3.10 (1.29–7.45)
Index case: unknown	1	0.36	2.76 (0.39–19.60)
			
*Cervical cancer (mothers and sisters)*			
Index case: BRCA1 positive	0	0.40	—
Index case: BRCA2 positive	0	0.30	—
Index case: negative	9	6.18	1.46 (0.76–2.80)
Index case: unknown	0	1.32	—
			
*Lung cancer (parents and siblings)*			
Index case: BRCA1 positive	1	0.17	5.95 (0.84–42.21)
Index case: BRCA2 positive	1	0.11	9.18 (1.29–65.20)
Index case: negative	16	2.07	7.73 (4.74–12.62)
Index case: unknown	2	0.50	4.00 (1.00–16.00)
			
*Stomach cancer (parents and siblings)*			
Index case: BRCA1 positive	0	0.60	—
Index case: BRCA2 positive	0	0.45	—
Index case: negative	8	7.77	1.03 (0.52–2.00)
Index case: unknown	0	1.61	
			
*Urinary cancers (parents and siblings)*			
Index case: BRCA1 positive	0	0.09	—
Index case: BRCA2 positive	0	0.06	—
Index case: negative	5	1.15	4.35 (1.81–10.46)
Index case: unknown	2	0.28	7.23 (1.81–28.90)

Abbreviations: CI=confidence interval; SIR=standardised incidence ratio.
