# Supplementary material for: Increased cancer risks for relatives of very early-onset breast cancer cases with and without BRCA1 and BRCA2 mutations
Source: Br J Cancer. 2010 Sep 7;103(7):1103–8. doi: 10.1038/sj.bjc.6605876 (PMC2965877; doi:10.1038/sj.bjc.6605876)
Supplement: Supplementary Material [file 6605876x1.doc]

**Supplementary Material**

*Subjects*

In San Francisco, eligible index cases diagnosed between 1995 and 1998 were identified through the Greater Bay Area Cancer Registry which ascertains all incident cancers as part of the Surveillance, Epidemiology and End Results program and the California Cancer Registry (John et al., 2004). In Ontario, eligible index cases diagnosed between 1996 and 1998 were identified through the Ontario Cancer Registry (Knight et al., 2002; John et al., 2004; Knight et al., 2004) while in Melbourne and Sydney, eligible index cases diagnosed between 1992 and 1999 and living in the metropolitan areas of Melbourne and Sydney were identified through the Victorian and New South Wales Cancer Registries respectively (McCredie et al., 1998; Hopper et al., 1999a; Dite et al., 2003; John et al., 2004).

*Mutation testing*

In San Francisco, 82 (80%) index cases were tested for mutations in BRCA1: 9 (11%) sequenced by Myriad Genetics Inc., 66 (80%) screened using 2-dimensional gel electrophoresis (Chen et al., 2002; van Orsouw et al., 1999), 6 (7%) screened using heteroduplex analysis (Korkko et al., 1998; Ganguly et al., Miron et al., 2000) and 1 (1%) screened only for the known Ashkenazi founder mutations. Additionally, 79 (77%) index cases were tested for mutations in BRCA2: 51 (65%) sequenced by Myriad Genetics Inc., 26 (33%) tested using heteroduplex analysis (Korkko et al., 1998; Ganguly et al., 1998; Miron et al., 2000) and 2 (3%) screened only for the known Ashkenazi founder mutations.

In Ontario, 87 (70%) index cases were tested for mutations in BRCA1 and BRCA2, first by testing women of Ashkenazi heritage for the three founder mutations, then testing all other index cases using a protein truncation test with 5’ DNA sequencing that covered the complete coding sequence of both genes (Ozelik et al., 1996; Ozelik et al., 1997; Andrulis et al., 2002).

In Melbourne and Sydney, 250 (90%) index cases were tested for mutations in BRCA1 and 248 (90%) were for mutations in BRCA2. Screening of the entire coding region of BRCA1 and BRCA2 was targeted at index cases with a strong family history of breast cancer. Of those tested for BRCA1 mutations, 26 (10%) were screened by Myriad Genetics Inc., 40 (16%) were sequenced as described elsewhere (Southey et al., 1999), 70 (28%) were screened using 2-dimensional gel electrophoresis (Chen et al., 2002; van Orsouw et al., 1999). The remaining 114 (46%), were screened for protein truncating mutations in exon 11 using the protein truncation test (Hopper et al., 1999b) and were screened for Ashkenazi founder mutations and duplication of exon 13 by specific PCR amplification and sequencing (Leong et al., 1999; Puget et al., 1999). Of the 248 index cases tested for mutations in BRCA2, 54 (22%) were screened by Myriad Genetics Inc., 12 (5%) were manually sequenced as described in Southey et al. (1999) and the remaining 182 (73%) were screened for protein truncating mutations in exons 10, 11 and 27 using the protein truncation test (Hopper et al., 1999b) and screened for Ashkenazi founder mutations by specific PCR amplification and sequencing (Leong et al., 2000).

In San Francisco, 11 (73%) of the 15 index cases with at least one mother or sister with breast cancer were tested for mutations in BRCA1 (1 by sequencing and 10 by 2-dimensional gel electrophoresis) and 10 (67%) were tested for mutations in BRCA2 (7 by sequencing and 3 by heteroduplex analysis). In Ontario, 13 (76%) of the 17 index cases with at least a mother or sister with breast cancer were tested for mutations in BRCA1 and BRCA2. In Melbourne and Sydney, 24 (96%) of the 25 index cases with at least a mother or sister with breast cancer were tested for mutations in BRCA1 and BRCA2.

*Imputation of missing family data*

To impute the missing data, recorded data from the index case and her relatives (including first- and second-degree relatives not included in these analyses) and summary information on ages and time intervals obtained from the whole data set were used to estimate the missing data for the index cases’ parents and siblings. For each missing data item, multiple estimates were made whenever possible and the final estimate was the mean of these. Date of birth was estimated to be the mean of: a sibling’s date of birth; earlier than a child’s date of birth by 28 years for women and 33 years for men; 28 years later than their mother’s date of birth; 33 years later than their father’s date of birth; for women, 4 years later than their spouse’s date of birth and for men, 4 years earlier than their spouse’s date of birth. The mean age at diagnosis for those with a cancer diagnosis and the mean number of years between diagnosis and death for those who were deceased were calculated from the non-missing data for nine broad categories of cancer site. These were used to estimate age at diagnosis and age at death.

Andrulis IL, Anton-Culver H, Beck J, Bove B, Boyd J, Buys S, Godwin A, Hopper J, Li F, Neuhausen SL, Ozcelik H, Peel D, Santella RM, Southey M, Van Orsouw NJ, Venter D, Vijg J and Whittemore AS for the Cooperative Family Registry for Breast Cancer Studies. Comparison of DNA- and RNA-based Methods for Detection of Truncating BRCA1 Mutations. Hum Mutat 2002; 20: 65-73.

Chen WY, Garber JE, Higham S, Schneider KA, Davis KB, Deffenbaugh AM, Frank TS, Gelman RS, Li FP. BRCA 1/2 testing in the community setting. J Clin Oncology 2002; 20: 4485-92.

Dite GS, Jenkins MA, Southey MC, Hocking JS, Giles GG, McCredie MR, Venter DJ, Hopper JL. Familial risks, early-onset breast cancer, and BRCA1 and BRCA2 germline mutations. J Natl Cancer Inst 2003; 95: 448-57.

Ganguly T, Dhulipala R, Godmilow L, Ganguly A: High throughput

fluorescence-based conformation-sensitive gel electrophoresis (F-CSGE)

identifies six unique BRCA2 mutations and an overall low incidence of BRCA2

mutations in high-risk BRCA1-negative breast cancer families. Hum Genet 1998; 102: 549-56.

Hopper JL, Chenevix-Trench G, Jolley D, Dite GS, Jenkins MA, Venter DJ, McCredie MRE, Giles GG. Design and analysis issues in a population-based case-control-family-study of the genetic epidemiology of breast cancer, and the Cooperative Family Registry for Breast Cancer Studies (CFRBCS). J Natl Cancer Inst Mongr 1999a; 26: 95-100.

Hopper JL, Southey MC, Dite GS, Jolley DJ, Giles GG, McCredie MRE, Easton DF, Venter DJ and the Australian Breast Cancer Family Study. Population-based estimate of the average age-specific cumulative risk of breast cancer for a defined set of protein truncating mutations in BRCA1 and BRCA2. Cancer Epidemiology, Biomarkers and Prevention 1999b; 8: 741 – 747.

John EM, Hopper JL, Beck JC, Knight JA, Neuhausen SL, Senie RT, Ziogas A, Andrulis IL, Anton-Culver H, Boyd N, Buys SS, Daly MB, O'Malley FP, Santella RM, Southey MC, Venne VL, Venter DJ, West DW, Whittemore AS, Seminara D; Breast Cancer Family Registry. The Breast Cancer Family Registry: an infrastructure for cooperative multinational, interdisciplinary and translational studies of the genetic epidemiology of breast cancer. Breast Cancer Res 2004; 6: R375-89.

Knight JA, Sutherland HJ, Glendon G, Boyd NF, Andrulis IL for the Ontario Cancer Genetics Network. Characteristics associated with participation at various stages at the Ontario site of the cooperative family registry for breast cancer studies. Ann Epidemiol 2002; 12: 27-33.

Knight JA, Onay UV, Wells S, Li H, Shi EJQ, Andrulis IL, Ozelic H. Genetic variants of GPX1 and SOD2 and breast cancer risk at the Ontario site of the Breast Cancer Family Registry. Cancer Epidemiol Biomarkers Prev 2004; 13: 146-9.

Korkko, J., Annunen, S., Pihlajamaa, T., Prockop, D.J., and Ala-Kokko, L.

Conformation sensitive gel electrophoresis for simple and accurate detection of mutations: Comparison with denaturing gradient gel electrophoresis and

nucleotide sequencing. Proc Natl Acad Sci USA 1998; 95: 1681-5.

Leong T, Whitty J, Keilar M, Mifsud S, Ramsay J, Birrell G, Venter D, Southey M, McKay M. Mutation analysis of BRCA1 and BRCA2 cancer predisposition genes in radiation hypersensitive cancer patients. Int J Radiat Oncol Biol Phys 2000 48: 959-65.

McCredie MRE, Dite GS, Giles GG, Hopper JL. Breast cancer in Australian women under the age of 40. Cancer Causes Control 1998; 9: 189-98.

Miron A, Schildkraut JM, Rimer BK, Winer EP, Sugg Skinner C, Futreal PA, Culler D, Calingaert B, Clark S, Kelly Marcom P, Iglehart JD: Testing for hereditary breast and ovarian cancer in the southeastern United States. Ann Surg 2000; 231: 624-34.

Ozcelik H, Antebi YJ, Cole DE, Andrulis IL. Heteroduplex and protein truncation analysis of the BRCA1 185delAG mutation. Hum Genet 1996; 98: 310-2

Ozcelik H, Schmocker B, Di Nicola N, Shi XH, Langer B, Moore M, Taylor BR, Narod SA, Darlington G, Andrulis IL, Gallinger S, Redston M. Germline BRCA2 6174delT mutations in Ashkenazi Jewish pancreatic cancer patients. Nat Genet 1997; 16: 17-8.

Puget N, Sinilnikova OM, Stoppa-Lyonnet D, Audoynaud C, Pagès S, Lynch HT, Goldgar D, Lenoir GM, Mazoyer S. An Alu-mediated 6-kb duplication in the BRCA1 gene: a new founder mutation? Am J Hum Genet 1999; 64: 300-302.

Southey MC, Tesoriero AA, Andersen CR, Jennings KM, Brown SM, Dite GS, Jenkins MA, Osborne RH, Maskiell JA, Porter L, Giles GG, McCredie MRE, Hopper JL and Venter DJ. BRCA1 mutations and other sequence variants in a population-based sample of Australian women with breast cancer. British Journal of Cancer 1999; 79: 34-39.

van Orsouw NJ, Dhanda RK, Elhaji Y, Narod SA, Li FP, Eng C, Vijg J.

A highly accurate, low cost test for BRCA1 mutations. J Med Genet 1999;

36: 747-53.

**Supplementary Table 1. Risk of Verified Breast Cancer for Mothers and Sisters of Index Cases**

|  | **Observed** | **Expected** | **SIR (95% CI)** |
| --- | --- | --- | --- |
| Mothers & Sisters Combined | 37 | 11.83 | 3.13 (2.27 – 4.32) |
| Mothers Sisters | 29  8 | 10.41  1.42 | 2.79 (1.94 – 4.01)  5.63 (2.81 – 11.25) |
| San Francisco Ontario Melbourne & Sydney | 6  11  20 | 3.15  2.86  5.83 | 1.91 (0.86 – 4.25)  3.85 (2.13 – 6.96)  3.43 (2.21 – 5.32) |
| Mothers & Sisters of BRCA1 Positive Index Cases Mothers & Sisters of BRCA2 Positive Index Cases Mothers & Sisters of BRCA1 and BRCA2 Negative Index Cases Mothers & Sisters of BRCA1 and BRCA2 Untested Index Cases | 5  3  23  6 | 0.61  0.40  8.92  1.91 | 8.21 (3.42 – 19.72)  7.51 (2.42 – 23.29)  2.58 (1.71 – 3.88)  3.14 (1.41 – 6.98) |
| Mothers of BRCA1 and BRCA2 Negative Index Cases Sisters of BRCA1 and BRCA2 Negative Index Cases | 19  4 | 7.77  1.16 | 2.45 (1.56 – 3.84)  3.46 (1.30 – 9.21) |

**Supplementary Table 2. Risk of Verified Non-Breast Cancer* for Parents and Siblings of Index Cases**

|  | **Observed** | **Expected** | **SIR (95% CI)** |
| --- | --- | --- | --- |
| Parents and Siblings | 62 | 72.44 | 0.86 (0.67 – 1.10) |
| Fathers and Brothers Mothers and Sisters | 36  26 | 38.15  34.29 | 0.94 (0.68 – 1.31)  0.76 (0.52 – 1.11) |
| Parents Siblings | 51  11 | 62.34  10.10 | 0.82 (0.62 – 1.08)  1.09 (0.60 – 1.97) |
| Fathers Mothers Brothers Sisters | 32  19  4  7 | 33.05  29.30  5.11  4.99 | 0.97 (0.68 – 1.37)  0.65 (0.41 – 1.02)  0.78 (0.29 – 2.09)  1.40 (0.67 – 2.94) |
| San Francisco Ontario Melbourne and Sydney | 6  11  45 | 16.49  17.10  38.86 | 0.36 (0.16 – 0.81)  0.64 (0.36 – 1.16)  1.16 (0.86 – 1.55) |
| Index Case: BRCA1 Positive Index Case: BRCA2 Positive Index Case: Negative Index Case: Untested | 5  4  43  10 | 3.94  2.75  54.46  11.40 | 1.27 (0.53 – 3.05)  1.45 (0.55 – 3.87)  0.79 (0.59 – 1.06)  0.88 (0.47 – 1.63) |
| Index Case: Negative – Fathers and Brothers Index Case: Negative – Mothers and sisters | 25  18 | 28.40  22.06 | 0.88 (0.59 – 1.30)  0.69 (0.44 – 1.10) |
| Index Case: Negative – Parents Index Case: Negative – Siblings | 34  9 | 46.33  8.13 | 0.73 (0.52 – 1.03)  1.11 (0.58 – 2.13) |
| Index Case: Negative – Fathers Index Case: Negative – Mothers Index Case: Negative – Brothers Index Case: Negative – Sisters | 22  12  3  6 | 24.34  21.99  4.06  4.07 | 0.90 (0.60 – 1.37)  0.55 (0.31 – 0.96)  0.74 (0.24 – 2.29)  1.48 (0.66 – 3.28) |

* excluding non-melanoma skin cancer

**Supplementary Table 3. Risk of Verified Specific Cancers for Parents and Siblings of Index Cases**

|  | **Observed** | **Expected** | **SIR (95% CI)** |
| --- | --- | --- | --- |
| Ovarian Cancer (Mothers & Sisters)  Index Case: BRCA1 Positive  Index Case: BRCA2 Positive  Index Case: Negative  Index Case: Unknown  Overall | 2  0  2  0  4 | 0.16  0.11  2.45  0.54  3.25 | 12.38 (3.10 – 49.51)  –   0.82 (0.20 – 3.27)  –  1.23 (0.46 – 3.28) |
| Prostate Cancer (Fathers & Brothers)  Index Case: BRCA1 Positive  Index Case: BRCA2 Positive  Index Case: Negative  Index Case: Unknown  Overall | 0  2  5  1  8 | 0.13  0.11  1.33  0.26  1.84 | –   18.55 (4.64 – 74.17)  3.75 (1.56 – 9.01)  3.84 (0.54 – 27.24)  4.36 (2.18 – 8.72) |
| Colorectal Cancer (Parents & Siblings)  Index Case: BRCA1 Positive  Index Case: BRCA2 Positive  Index Case: Negative  Index Case: Unknown  Overall | 1  0  6  2  9 | 0.74  0.49  10.77  2.09  14.07 | 1.35 (0.19 – 9.57)  –   0.56 (0.25 – 1.24)  0.96 (0.24 – 3.83)  1.64 (0.33 – 1.23) |

| Brain Cancer (Parents and Siblings)  Index Case: BRCA1 Positive  Index Case: BRCA2 Positive  Index Case: Negative  Index Case: Unknown  Overall | 0  0  4  1  4 | 0.11  0.07  1.61  0.36  1.61 | –   –   2.48 (0.93 – 6.61)  –  2.48 (0.93 – 6.61) |
| --- | --- | --- | --- |
| Cervical Cancer (Mothers & Sisters)  Index Case: BRCA1 Positive  Index Case: BRCA2 Positive  Index Case: Negative  Index Case: Unknown  Overall | 0  0  2  0  2 | 0.40  0.30  6.18  1.32  8.19 | –  –  0.32 (0.08 - 1.29)  –  0.24 (0.06 – 0.98) |
| Lung Cancer (Parents & Siblings)  Index Case: BRCA1 Positive  Index Case: BRCA2 Positive  Index Case: Negative  Index Case: Unknown  Overall | 1  1  4  1  7 | 0.17  0.11  2.07  0.50  2.84 | 5.95 (0.84 – 42.21)  9.18 (1.29 – 65.20)  1.93 (0.73 – 5.15)  4.00 (0.28 – 14.20)  2.46 (1.17 – 5.17) |

| Stomach Cancer (Parents & Siblings)  Index Case: BRCA1 Positive  Index Case: BRCA2 Positive  Index Case: Negative  Index Case: Unknown  Overall | 0  0  3  0  3 | 0.60  0.45  7.77  1.61  10.41 | –  –  0.39 (0.12 – 1.20)  –  0.29 (0.09 – 0.89) |
| --- | --- | --- | --- |
| Urinary Cancers (Parents & Siblings)  Index Case: BRCA1 Positive  Index Case: BRCA2 Positive  Index Case: Negative  Index Case: Unknown  Overall | 0  0  3  2  5 | 0.09  0.06  1.15  0.28  1.57 | –  –  2.61 (0.84 – 8.10)  7.23 (1.81 – 28.90)  3.18 (1.32 – 7.64) |
